# Bioinformatic Analysis of the Effect of the *Sirtuin* Family on Differentiated Thyroid Carcinoma

**DOI:** 10.1155/2022/5794118

**Published:** 2022-01-30

**Authors:** Lijun Yao, Yinhua Wang

**Affiliations:** Department of Oncology, Suzhou Ninth People's Hospital, Suzhou 215200, China

## Abstract

A growing body of experimental evidence suggests that *sirtuins* (*SIRTs*) are associated with tumorigenesis in differentiated thyroid cancer (DTC). Nevertheless, the involvement of *SIRTs* in the pathogenesis of DTC and their clinical value remain ill-defined and should be thoroughly examined. We explored the transcription of SIRTs and survival data of patients with DTC by the systematic utilization of bioinformatics to analyze data of publicly accessible databases including Oncomine, cBioPortal, Kaplan-Meier Plotter, Gene Expression Profiling Interactive Analysis (GEPIA), Protein Atlas, LinkedOmics, and GSCALite. The examination of gene expression profiles showed that *SIRT2*, *SIRT3*, *SIRT4*, *SIRT5*, and *SIRT6* were downregulated in DTC tissues compared with the normal thyroid tissues. The decreased expression levels of *SIRT2*, *SIRT4*, and *SIRT5* were correlated with advanced tumor stages. The survival results showed that the increased *SIRT4* mRNA expression level was associated with improved overall survival (OS) in the DTC patients. In addition, patients with DTC with high *SIRT2*, *SIRT3*, *SIRT4*, and *SIRT5* mRNA levels had higher disease-free survival (DFS). These results showed that *SIRT2*, *SIRT3*, *SIRT4*, *SIRT5*, and *SIRT6* are potential targets for precise treatment of DTC patients and that *SIRT2*, *SIRT3*, *SIRT4*, and *SIRT5* are novel potential biomarkers for the prognosis of DTC.

## 1. Introduction

Thyroid cancer is classified among the most widespread malignant tumors that occur in the endocrine system. The global incidence of thyroid cancer, especially differentiated thyroid cancer (DTC), has been steadily amplified in recent years. DTC is the major type of thyroid cancer and encompasses papillary thyroid cancer (PTC) and follicular thyroid cancer (FTC) [1]. PTC accounts for about 80% of thyroid cancer, while FTC accounts for about 15% within the total of 95% DTC patients [2]. Currently, the main treatments for DTC include surgery, postoperative assisted ablation, thyroid stimulating hormone (TSH) inhibition therapy, and targeted molecular therapy [3]. Targeted molecular therapy has become a new approach for the treatment of advanced thyroid cancer [4, 5]. Studies have shown that *RET*, *RAS*, *BRAF*, and *VEGF* are closely related to the pathogenesis of thyroid cancer, which lay a foundation for targeted molecular therapy [6–8]. About 70% of PTC is caused by *BRAF* mutations, *RAS* mutations and *RET/PTC* gene rearrangements [9]. In FTC, *RAS* point mutations, and *PPARγ/PAX8* gene rearrangements produce the *PPFP* fusion gene, the most common oncogene alteration, and *PTEN* deletion/mutation; *PIK3CA* mutation and *IDH1* mutation are also responsible for FTC [10–13]. Small molecule inhibitors targeting these signaling kinases have become a hot spot for targeted therapies. Due to the heterogeneity of tumors, the use of biomarkers for predicting targeted therapies has some limitations. Therefore, new biomarkers are needed in this field to effectively enhance prognosis and individualized treatment.

*Sirtuins* (*SIRTs*) are deacetylases that are highly conserved from bacteria to humans. To date, there are seven recognized members of the human *SIRT* family which are numbered in order of their discovery into *SIRT1-7*. X-ray crystal diffraction revealed that multiple members of the *SIRT* family contain one small domain made of approximately 270 amino acids and a large domain [14]. The large domain is mainly constituted of the Rossmann folds while the small domain encompasses a zinc finger structure [15]. The *SIRT* family has a deacetylase activity and ADP-nuclease transferase activity, and the deacetylation mediated by *SIRTs* is characterized by the transfer of the acetyl group to the ADP-ribosyl of NAD [16]. *SIRTs* mediate both catalytic activities of deacetylation and NAD cleavage. The ADP-ribosyltransferase activity of *SIRTs* is the transfer of ADP-ribose from NAD to acetylated proteins [16]. *SIRTs* are of great importance in clinical medicine and basic research. *SIRTs* are significantly dysregulated in many malignancies challenging human health, in particular colorectal cancer, prostate cancer, ovarian cancer, lung cancer, breast cancer, and thyroid cancer [16–19]. The interactions between mammalian *SIRTs* and *FOXO/PGC-1α*, *Ku70*, *NF-κB*, *p53*, and other proteins modulate cellular metabolism, cellular stress response, aging, and apoptosis [20, 21]. *SIRTs* are thought to have complex and unique features in human DTC [10, 11]. *SIRT1* was shown to participate in the regulation of *p21* and *Bax*-related molecular events via the *SIRT1-Foxp3* pathway in PTC cells [22]. Other research groups have found that by inhibiting *ERK* and *Mcl-1*, *SIRT6* silencing can downregulate the invasiveness of PTC cells in vitro. Compared with normal thyroid cancer cells, the expression of *SIRT7* was significantly increased in DTC, and the overexpression of *SIRT7* and *SIRT1* conferred resistance to DTC cells [23, 24]. In addition, some researchers have found that the *SIRT* family plays a pivotal role by downregulating the expression pattern of the tumor-suppressor gene *ARHI* in thyroid cancer cells [25]. But so far, which *SIRT* family is activated or inhibited and the unique function of *SIRTs* in thyroid cancer remain to be absolutely deciphered [16]. Dysregulation of the *SIRTs* and its relationship with clinical and pathological traits and the predictive value have been conveyed in human thyroid cancer. With the advent of microarray and next-generation sequencing technology, revolutionary advances have become an important part of biological and biomedical research [26, 27] and have allowed data mining using bioinformatical approaches. Nevertheless, to the best of our knowledge, bioinformatical approaches have not been used to figure out the link between the *SIRTs* and DTC [28].

Herein, based on publicly available databases, we analyzed in detail different *SIRTs* in patients with DTC to examine their expression changes, probable function, and prognostic value of *SIRT* family in DTC.

## 2. Materials and Methods

### 2.1. Patients

This study was performed based on bioinformatics analysis of The Cancer Genome Atlas (TCGA) data stored in different databases, namely Oncomine, cBioPortal, Kaplan-Meier Plotter, The Gene Expression Profiling Interactive Analysis (GEPIA), Protein Atlas, LinkedOmics, and GSCALite. No particular approval was needed, and the study followed TCGA policies.

### 2.2. Oncomine

Oncomine is a database containing microarray expression data for cancers and integrated data-mining platform (http://www.oncomine.org/). Oncomine was employed for analyzing and visualizing the expression levels of genes in the *SIRT* family members in diverse cancers following the online instructions. The mRNA levels of *SIRTs* in normal and cancer tissues were compared, and Student's *t* test was used for assessing the difference between both groups. The significant differences were declared at *p* < 0.05.

### 2.3. GEPIA

GEPIA is an interactive online platform for mining the RNA sequencing data from the Genotype-Tissue Expression (GTEx) and TCGA projects (http://gepia.cancer-pku.cn/). GEPIA was used for analyzing the expression profiles of *SIRTs* in DTC and its pathological stages following the default settings online. GEPIA was also used for survival analysis based on the *SIRTs*.

### 2.4. The Kaplan-Meier Plotter

The Kaplan-Meier Plotter (http://www.kmplot.com/) was used to analyze the OS and DFS of patients with DTC. The samples were grouped into high expression and low expression groups relatively to the median expression. The Kaplan-Meier Plotter was used for generating the survival plot containing the log rank *p* value and the hazard ratio (HR) with 95% confidence intervals (CIs).

### 2.5. cBioPortal

TCGA database contains genomic and clinical data on more than 30 cancer types. Samples from TCGA-THCA dataset were chosen and used for the analysis of *SIRTs* using cBioPortal (http://www.cbioportal.org). Genetic variations were analyzed by selecting copy number alterations (CNAs) and mutations as selected molecular profiles.

### 2.6. Human Protein Atlas

Immunostaining images of *SIRTs* in human DTC tissues compared to nontumorous thyroid tissues were obtained from the Protein Atlas database (https://www.proteinatlas.org).

### 2.7. LinkedOmics

The LinkedOmics database is a multiomics tool for the interpretation of attribute associations between the existing cancer databases [29]. It was used for association analysis between the *SIRTs* and other genes in the DTC RNA-Seq data. The analysis was performed online following the instructions displayed on the platform (http://www.linkedomics.org).

### 2.8. Functional Enrichment Analysis

GO and KEGG functional enrichment analyses were performed to uncover the functions prominently associated with the *SIRTs* and their coregulated genes using the ClusterProfiler package in the R software.

## 3. Results

### 3.1. Genomic Profiles of SIRTs

**Figure 1:**
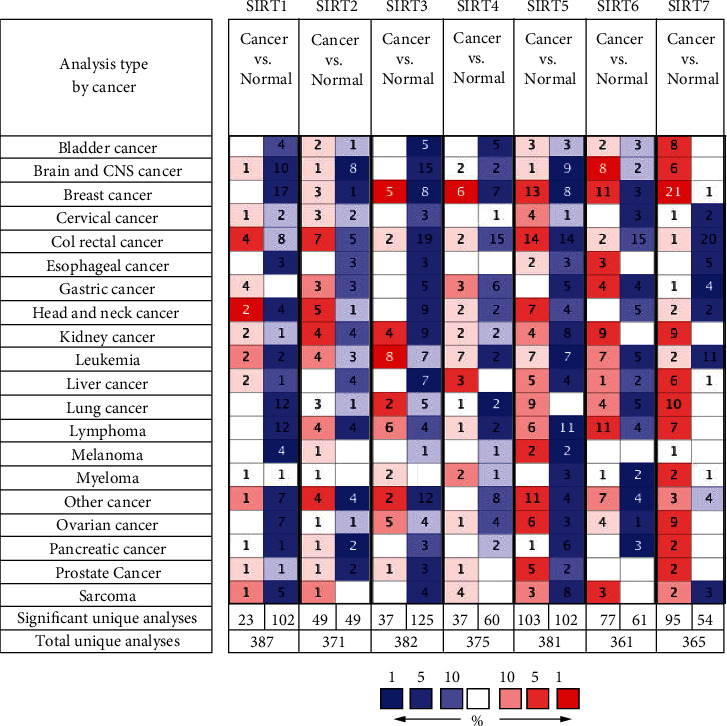
The transcription levels of *SIRTs* in different types of cancers (Oncomine). Red color represents elevated expression, and blue color represents reduced expression. The depth of the color represents the best gene rank percentile.

The genomic profiles of *SIRT* family members in patients with DTC were assessed utilizing the Oncomine, GEPA, and Human Protein Atlas databases. The transcriptional expression of *SIRTs* between various normal and cancer tissues was explored by data analysis in the Oncomine database (Figure 1). The results indicated that *SIRTs* were diversely expressed in different cancer types. Here, DTC was classified to head and neck cancer. Six unique analyses showed significant differences for *SIRT1* in head and neck cancer, of which two were upregulated and four were downregulated. Similar results were observed for *SIRT3* and *SIRT4*. The same expression trends were also observed in the other cancer types. The expression levels of *SIRT2* in head and neck cancer were upregulated in five unique analyses and downregulated in one unique analysis. Similar results were observed for *SIRT5*, including 7 upregulations and 4 downregulations. Compared with normal tissues, *SIRT6* mRNA expression levels in head and neck cancer were significantly decreased in five unique analyses. The expression levels of *SIRT7* were elevated in the majority of analyses across all types of cancer. For head and neck cancer, however, SIRT7 was upregulated in two unique analyses and downregulated in two unique analyses.

**Figure 2:**
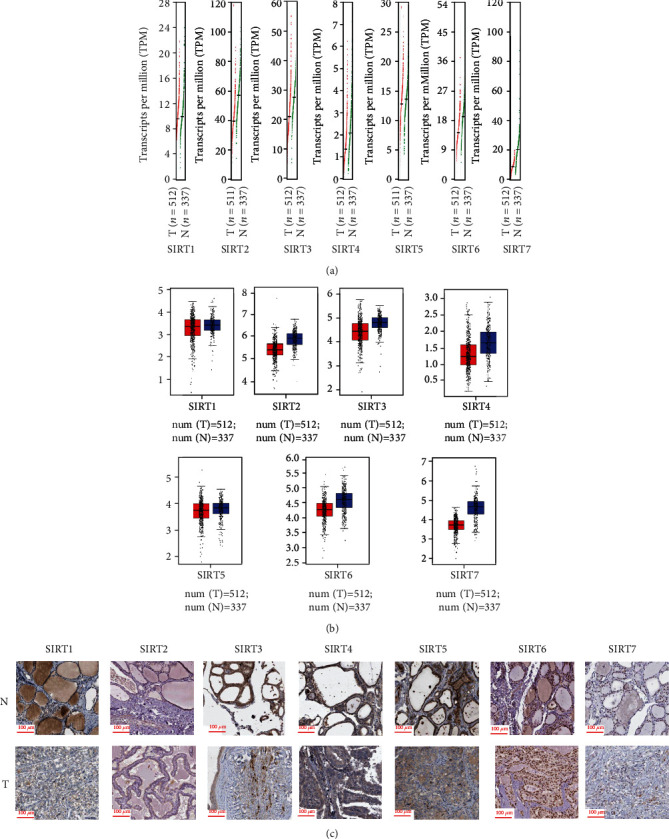
The expression of *SIRT* family members in DTC using GEPIA. (a) Scatter diagram showing the expression profile of *SIRTs* in DTC and normal samples. Red color represents the *SIRT* expression level in tumor samples, and green color represents the *SIRT* expression level in normal samples. (b) Box plot showing the expression of *SIRTs* in DTC and normal samples. Red color represents the *SIRT* expression level in tumor samples, and blue color represents the *SIRT* expression level in normal samples. (c) Representative IHC images of *SIRTs* in DTC. T represents tumor tissues and N represents normal tissues. Positive staining was mainly concentrated at the nucleus.

**Figure 3:**
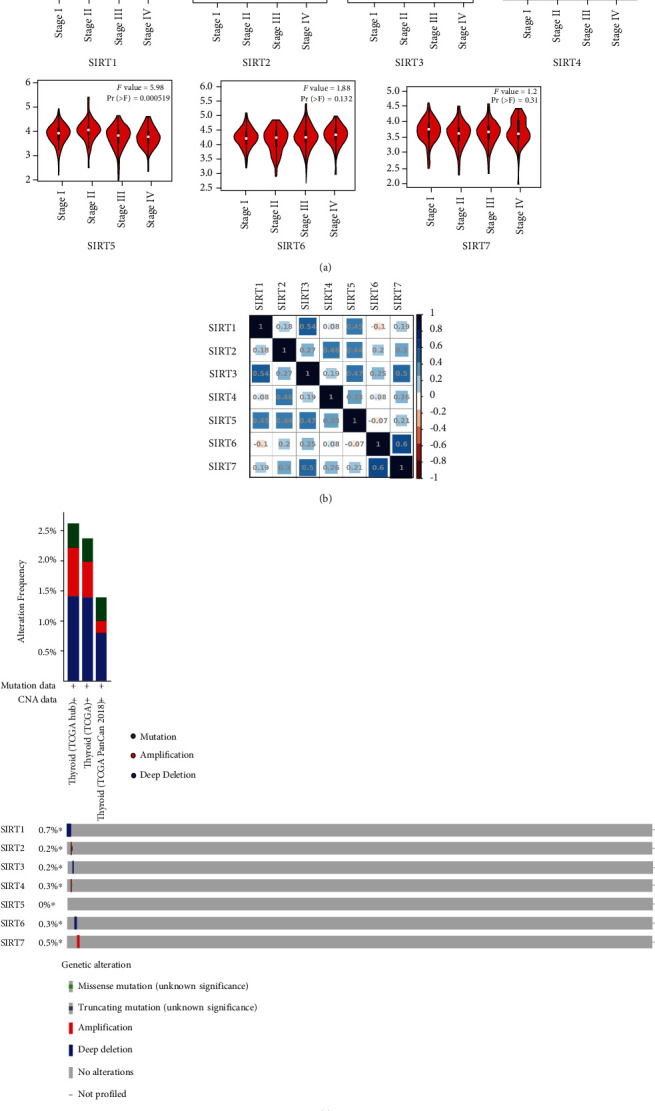
Correlation between *SIRT* expression and tumor stage in DTC (GEPIA) and genetic variation. (a) Association between the expression level of *SIRTs* and tumor stages. (b) Correlation analysis between *SIRTs* in DTC. Darker colors represent the higher correlation. (c) Genetic variation of *SIRTs* in DTC. Genetic variation included mutation, amplification, and deep deletion.

Next, the GEPIA database was exploited for examining the expression of the *SIRTs* in DTC samples in comparison with the normal tissues at mRNA level. The scatter diagram and the box plot of the expression levels of *SIRTs* are reported in Figures 2(a) and 2(b), respectively. 512 DTC samples and 337 normal tissues were selected. The results displayed that the expression levels of *SIRT2*, *SIRT3*, *SIRT4*, *SIRT6*, and *SIRT7* in DTC tissues were lower than those in normal tissues (Figures 2(a) and 2(b)). Immunohistochemistry (IHC) analysis from the Protein Atlas database was done to assess the protein expression of *SIRT* proteins in DTC tissues. There was moderate or weak immunoreactivity of *SIRTs 1*, *3*, *4*, *5*, *6*, and *7* in DTC tissues while their corresponding immunoreactivity was relatively weaker in normal tissues (Figure 2(c)). *SIRT2* was not detected in both DTC and normal tissues. We also analyzed the expression of the *SIRT* family in different tumor stages in DTC. The decreased expression of *SIRT2*, *SIRT4*, and *SIRT5* was significantly associated with advanced stages of DTC, while the expression levels of *SIRT1*, *SIRT3*, *SIRT6*, and *SIRT7* groups did not differ significantly between the DTC stages (Figure 3(a)). Pearson's correlation was also performed to evaluate whether there was a relationship between *SIRTs* in DTC. The results showed that there were significantly positive correlations observed between *SIRT1* and *SIRT2/3/5/7*, *SIRT2* and *SIRT3/4/5/6/7*, *SIRT3* and *SIRT4/5/6/7*, *SIRT4* and *SIRT5/7*, *SIRT5* and *SIRT7*, and *SIRT6* and *SIRT7* (Figure 3(b), shown in blue). *SIRT1* had a negative correlation with *SIRT6* (*p* = 0.028). No significant correlations were found between *SIRT1* and *SIRT4*, *SIRT4* and *SIRT6*, or *SIRT5* and *SIRT6*. 32 (2%) of selected patients (1503) had altered genes, including missense mutation (*SIRT4* and *SIRT6*), truncating mutation (*SIRT2*), amplification (*SIRT2*, *SIRT4*, and *SIRT7*), and deep deletion (*SIRT1*, *SIRT3*, and *SIRT6*, Figure 3(c)). No alteration was recorded for *SIRT5*.

### 3.2. Prognosis Evaluation of SIRTs in DTC Patients

**Figure 4:**
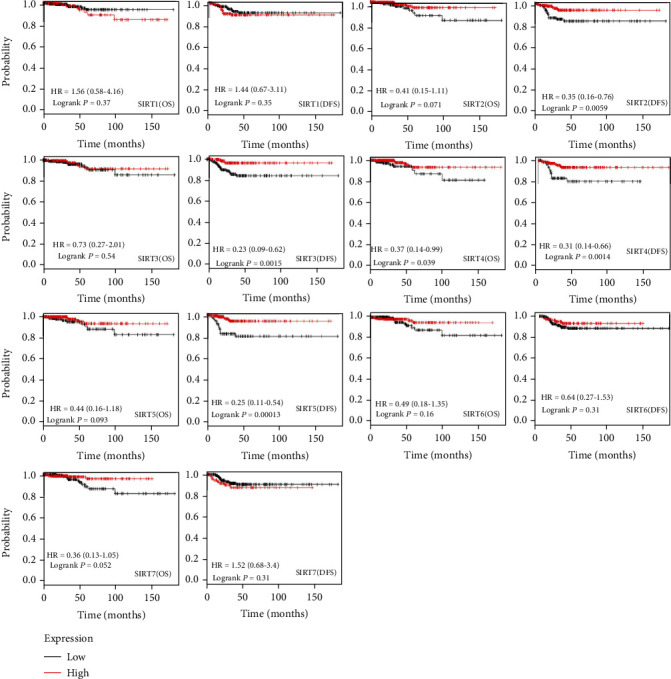
The prognostic value of mRNA level of *SIRT* factors in DTC (Kaplan-Meier Plotter), including OS and DFS analysis. HR > 1.0 represents a risky gene, and HR < 1.0 represents a protective gene.

To explore the possible involvement of the *SIRT* family in the survival outcomes of DTC patients, the Kaplan-Meier Plotter tool was utilized to assess their survival rates by using the openly accessible DTC datasets. A positive correlation between the increased *SIRT4* mRNA expression level with improved overall survival (OS) was recorded (Figure 4, *p* < 0.05). The increased *SIRT7* mRNA expression had a trend toward better OS (*p* = 0.052). DTC patients with high *SIRT2*, *SIRT3*, *SIRT4*, and *SIRT5* mRNA levels had longer disease-free survival (DFS) time (Figure 4, *p* < 0.001).

### 3.3. Identification of Genes Correlated with SIRTs

**Figure 5:**
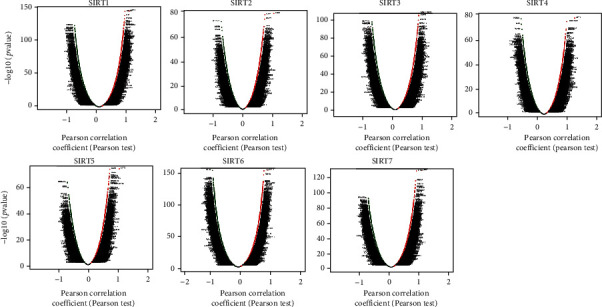
Volcano plot of association results showing the correlation of different *SIRTs* with gene expression in DTC (LinkedOmics). Red dots represent positive correlation, and green dots represent negative correlation.

**Figure 6:**
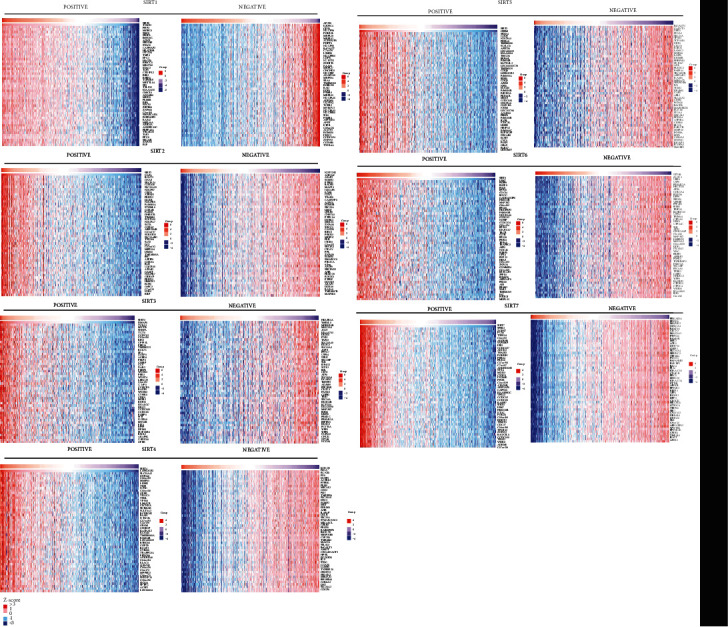
Heatmap plot of association results showing the correlation of different *SIRTs* with gene expression in DTC (LinkedOmics). Top 50 genes with the greatest correlation were presented.

With the aim of identifying the genes that were correlated with the expression of *SIRTs*, the Pearson correlation analysis was performed using the LinkedOmics database. The results indicated the expression of *SIRTs* was significantly correlated with a multitude of genes (Figures 5 and 6). We found that *TNKS2*, *PHF3*, *MORC3*, *SMC3*, and *SPOPL* were genes most positively correlated with *SIRT1* while *AP2S1*, *EXOSC4*, *GPS1*, *NDUFS8*, and *POLR2L* were the genes most negatively associated with *SIRT1* (Figures 5 and 6). Genes such as *NAPA*, *BCAT2*, and *GNAS* were the most positively correlated with *SIRT2* while *NOTCH2*, *AHNAK*, *ASAP2*, and *SGMS2* were genes most negatively associated with *SIRT2* (Figures 5 and 6). Genes positively associated with *SIRT3* were represented by *DMAP1*, *TCEA2*, *MYST1*, and *SNRPA* whereas the most negatively correlated genes were *HEATR5A* and *YME1L1* (Figures 5 and 6). *SLC25A42*, *ZNF346*, and *LOH12CR2* were genes most positively associated with *SIRT5* while *KCNQ3*, *FLNA*, *RUNX1*, and *FN1* were those most significantly and negatively associated with *SIRT4* (Figures 5 and 6). The genes most positively correlated with *SIRT5* were *OXSM*, *SFXN4*, *COQ9*, and *NDUFA5* while those most negatively regulated with this gene were *B4GALT5* and *GALNT5* (Figures 5 and 6). *SIRT6* was most positively associated with *LSM7* and *FKBP8* but most negatively associated with *STT3B* and *CLCN3* while genes most positively associated with *SIRT7* were *SPSB3*, *ZGPAT*, *PUS1*, and *TSEN54* but *PRKAR2A* and *PDZD8* were the most negatively associated with *SIRT7* (Figures 5 and 6). Specially, *BRAF* mutation is the most common gene alteration in DTC [30]. We examined the correlation between *SIRTs* and *BRAF* and found that *BRAF* was positively associated with *SIRT1*, *SIRT2*, *SIRT3*, *SIRT4*, *SIRT5*, and *7* but negatively associated with *SIRT6* (*p* < 0.05).

### 3.4. Functional Analysis of SIRTs

**Figure 7:**
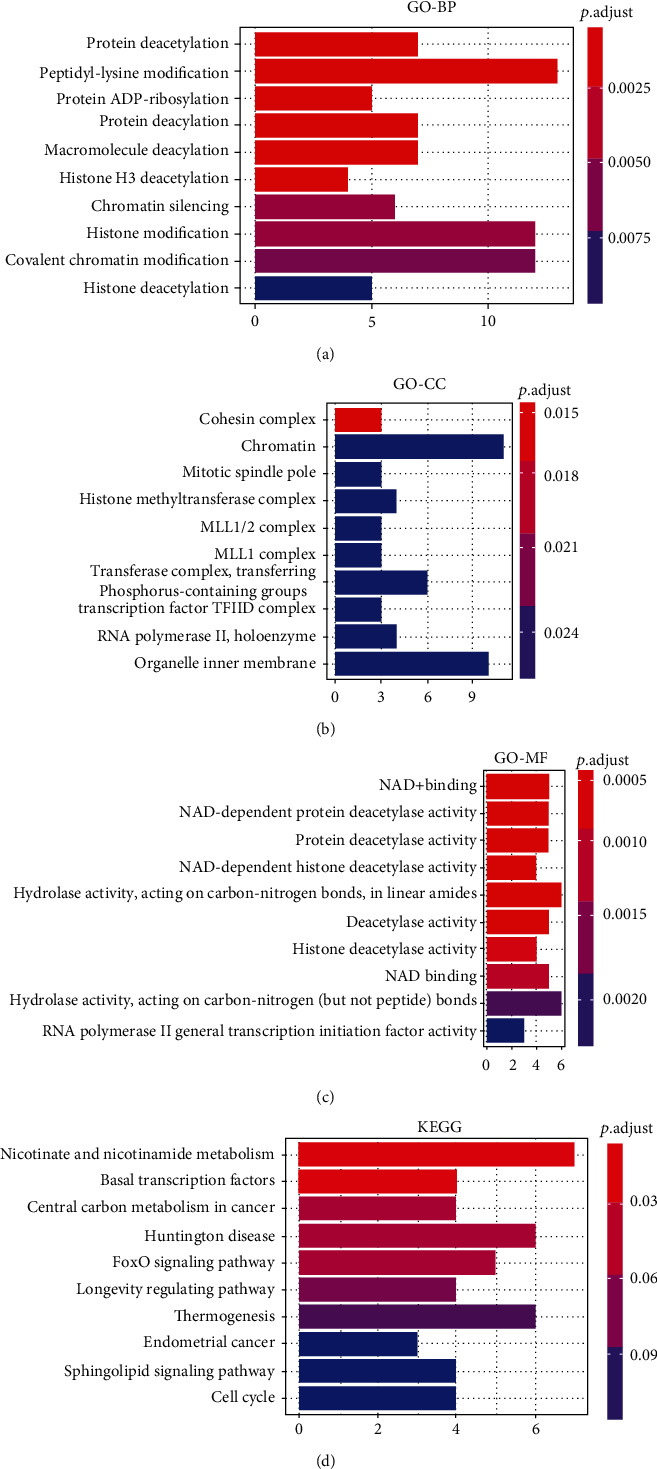
Functional enrichment analysis of *SIRTs* and coregulated genes in DTC. (a) Biological process. (b) Cellular component. (c) Molecular function. (d) KEGG pathways. The intensity of the colors represents the *p* value (the redder the color, the lower *p* value). Top 10 significant terms were presented.

**Figure 8:**
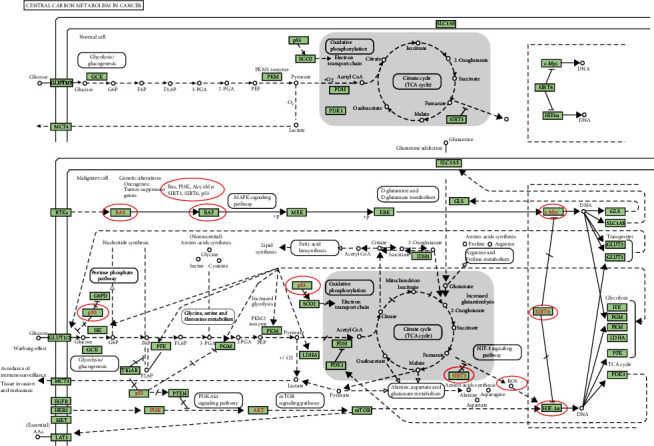
Carbon metabolism in cancer pathway. *SIRTs* and coregulated genes are noted in a red circle. Solid arrow represents epicardial activation; dashed arrow represents septal activation; T-shaped arrow represents inhibition.

**Figure 9:**
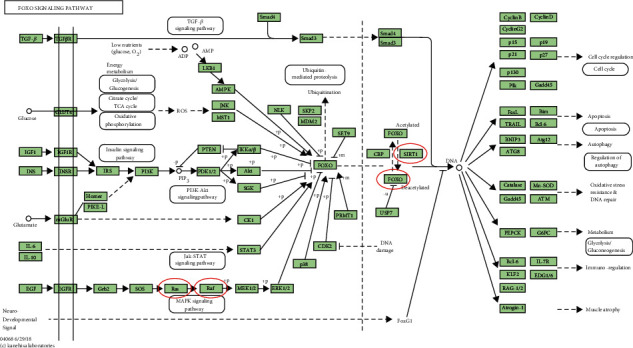
The FOXO signaling pathway. *SIRTs* and coregulated genes are noted in a red circle. Solid arrow represents epicardial activation; dashed arrow represents septal activation; T-shaped arrow represents inhibition.

In order to uncover the functions prominently associated with the *SIRTs* and their coregulated genes, functional enrichment analysis was performed on a set of genes containing *SIRTs* and genes correlated with *SIRTs* with *r* greater than 0.8 and *p* < 0.05. The results indicated that *SIRTs* and coregulated genes were involved in biological processes (BP) of protein deacetylation, peptidyl-lysine modification, protein ADP-ribosylation, and protein diacylation (Figure 7(a)). In the category of cellular component (CC), cohesin complex, chromatin, and mitotic spindle pole were the most represented gene ontology (GO) terms while in the category of molecular function (MF), NAD+ binding, NAD-dependent protein deacetylase activity, and protein deacetylase activity were the predominant GO terms (Figures 7(b) and 7(c)). The KEGG pathway enrichment analysis showed that nicotinamide and nicotinamide metabolism, basal transcription factors, central carbon metabolism in cancer, Huntington's disease, and FOXO signaling pathway were the most significantly enriched signaling pathways associated with *SIRTs* and their correlated genes in DTC (Figure 7(d)). In the pathway of central carbon metabolism in cancer, *SIRT6* could directly inhibit hypoxia-inducible factor 1*α* (*HIF-1α*), and *SIRT3* could inhibit *HIF-1α* by repressing *HIF-1* signaling, which then affected metabolic process such as glycolysis and tricarboxylic acid (TCA) cycle (Figure 8). The *Ras/Raf/ERK/MAPK* pathway was also regulated by *SIRT6*, thus contributing to *c-Myc* deregulated expression. In addition, *SIRT1*, together with *BARF*, could modulate the FOXO signaling pathway (Figure 9).

## 4. Discussion

Dysregulation of *SIRTs* has been investigated in diverse cancers [24]. Although the function and prognostic values of *SIRTs* had been partly validated in various cancers, no bioinformatics analysis of *SIRTs* has been performed in DTC [31, 32]. This study reported for the first time the mRNA expression profiles and clinical value of *SIRTs* in DTC. Our findings will help leverage existing knowledge to improve treatment design and improve the prognosis of patients with DTC.

*SIRT1* was generally known as an oncogene and involved in multiple cellular processes including cell cycle, apoptosis, and cancer metastasis [33]. *SIRT1* was acknowledged as a direct downstream target of *miR-212*, which hindered the proliferation and promoted the apoptosis of thyroid cancer cells by negatively regulating *SIRT1* [34]. It has also been reported that *SIRT1-Foxp3* signaling-mediated regulation of *Bax* and *p21* mRNA expression is a hallmark molecular event in DTC and shows significant resistance to genotoxic stress induced by the chemotherapeutic agent etoposide [22]. Li et al. [35] discovered that *SIRT7* could promote tumorigenesis by acting on the *DBC1/SIRT1* axis in PTC cells. The result was consistent with our findings that *SIRT1* and *SIRT7* were correlated significantly (*r* = 0.19, *p* < 0.001). Roehlen et al. [36] demonstrated that the *vitamin D-SIRT1-FOXO3a* axis played a pivotal role in DTC and Hashimoto thyroiditis. Our KEGG results also showed *SIRT1* affected the FOXO signaling pathway along with *BRAF*. In the study, however, no significant associations were found between *SIRT1* and clinical characteristics.

So far, our knowledge on the expression and regulation of *SIRT2* in DTC is limited. In contrast to previous results suggesting a broad tumor-promoting effect for *SIRT2* [37], our study indicated that *SIRT2* expression was decreased in DTC tissues compared to the nontumorous tissues. Its expression pattern was significantly associated with tumor stage. Higher expression of *SIRT2* was not significantly associated with OS but was correlated with improved DFS in all patients with DTC, which suggested *SIRT2* as a possible target of treatment.

*SIRT3*, a nicotinamide adenine dinucleotide- (NAD-) dependent deacetylase, was often recognized as a tumor-suppressor gene [38]. However, *SIRT3* was reported to be highly expressed in DTC compared to benign thyroid tumor and might involve mitochondrial alterations [39]. Wang et al. showed that *miR-1225-5p* could promote DTC cell proliferation and metastasis via targeting *SIRT3* directly [40]. These results and ours were not in accordance. Our findings indicated that the mRNA expression level of *SIRT3* was slightly lower in DTC tissue than in normal tissue, and patients with high DTC expression had better DFS. Additionally, there have been a few studies on *SIRT3/HIF-1α* pathway in cervical cancer and hepatocellular cancer but not in DTC [41–43]. More validation studies need to be performed to further investigate the role of *SIRT3* in DTC.

*SIRT4* displayed deacetylase activity and were involved in regulating cellular energy metabolism [44]. Studies showed that *SIRT4* was also reported to significantly decrease in thyroid cancer and inhibit glutamine metabolism and thus inhibit cell proliferation and invasion [45]. This is consistent with our predictions. In our study, we demonstrated that the expression of *SIRT4* in DTC tissues was downregulated, and its expression was associated with tumor progression. Moreover, low expression of *SIRT4* was markedly correlated with poor OS and DFS, which corroborated with the findings that *SIRT4* is an antitumor gene [46]. This suggests that *SIRT4* may be considered a potential biomarker of poor prognosis and an effective molecular target of treatment for DTC.

*SIRT5* played a very important role in fatty acid oxidation, glycolysis, TCA cycle, apoptosis, and antioxidant defense [47]. Some researchers also found that *SIRT5* was upregulated in cisplatin-resistant ovarian cancer cells compared with cisplatin-sensitive cells [48]. In addition, *SIRT5* was found to promote cisplatin resistance (HO-1) pathway in ovarian cancer by modulating Nrf2/heme oxygenase 1 axis [48]. However, no study has reported the role of *SIRT5* in DTC. In the present study, low expression of *SIRT5* was observed in DTC patients with advanced diseases and significantly correlated with improved DFS, suggesting *SIRT5* may function as a tumor-suppressor gene.

Previous works indicated that *SIRT6* plays a relevant role in aging biochemical functions involved in tumor progression and could constitute an antitumor therapeutic target [49]. Qu et al. [50] demonstrated that *SIRT6* enhanced their malignant behavior through the *BRAF/ERK/Mcl-1* pathway. Our studies also found the significant relationship between *SIRT6* and *BRAF* (Figures 3(b) and 8). Nevertheless, *SIRT6* seemed to have no effect on tumor stage or clinical outcomes.

*SIRT7* was upregulated in multiple cancers including DTC and could promote the tumorigenesis of DTC cells in vitro and in vivo [35, 51–53]. *SIRT7* is reported to be correlated with active rRNA genes (rDNA) and actively increases outgrowth and proliferation of U2OS cells [54]. Compared to the *SIRT7*-wildtype hepatoma cell line, the *SIRT7*-deficient cell line exhibited exquisite sensitivity to doxorubicin via the *SIRT7-P53-NOXA* axis [55]. Inhibition of *SIRT7* using small interfering RNAs inhibits tumor resistance to radiation [56, 57]. In the present study, *SIRT7* expression was not associated with tumor stage and prognosis in DTC.

Functional enrichment of *SIRTs* and their coregulated genes in DTC indicated that these genes were involved in protein deacetylation, peptidyl-lysine modification, protein ADP-ribosylation, and protein diacylation. The most significant pathways were nicotinamide and nicotinamide metabolism pathways and basal transcription factors. These results indicated that *SIRTs* may participate in the pathogenesis of DTC by regulating these pathways and biological processes. In NIH3T3 cells, *SIRT1* causes ubiquitination and degradation of *FOXO3*, a FOXO transcription factor family member that can play a crucial role in tumor suppression and metabolism and may act as oncogene [58]. Previous studies reported that *SIRT2* mediates the acetylation of pyruvate kinase to regulate tumor growth [59]. *SIRT3*, downregulated in cholangiocarcinoma (CCA) patients, can prevent tumor progression by inhibiting the *HIF1α/PDK1/PDHA1* pathway [60]. *SIRT5* was also found to modulate the deacetylation of *LDHB* and induce the autophagy in colorectal cancer [61]. Previous study showed that *SIRT6* might act as antioncogenesis factor by inhibiting *HIF-1α*, an angiogenesis-promoting molecule, in lung cancer [62], and by inhibiting *c-Myc* gene and ribosome biosynthesis [63]. Since our results showed decreased expression levels of *SIRT3* and *SIRT6* in DTC tissues, we estimate that *SIRT3* and *SIRT6* take effect through metabolic processes such as glycolysis and TCA cycle. Similarly, *SIRT7* was found to counteract cancer development by the deacetylation of *WDR77* [64]. These studies support our finding that the *SIRTs* and coregulated genes were involved in deacetylation in DTC. Moreover, our study corroborated with previous studies demonstrating that nicotinamide metabolism regulates cancer processes [65, 66].

## 5. Conclusion

In summary, we performed a systematic exploration to examine the expression profiles and clinical value of the *SIRT* family proteins in DTC and have provided an overview of these *SIRTs* in DTC. Our findings suggest that high expression of *SIRT2*, *SIRT3*, *SIRT4*, *SIRT5*, and *SIRT6* in DTC may indicate that they have significant regulatory functions in thyroid carcinogenesis. Therefore, *SIRT2*, *SIRT3*, *SIRT4*, *SIRT5*, and *SIRT6* may be relevant therapeutic targets for DTC. Moreover, the expression of *SIRT2*, *SIRT3*, *SIRT4*, and *SIRT5* may have potential as prognostic markers for determining the survival and prognosis of DTC.

## Data Availability

The datasets used in the present study can be obtained in The Cancer Genome Atlas (https://portal.gdc.cancer.gov/).
